# Health care resource utilization and direct medical costs for patients with schizophrenia initiating treatment with atypical versus typical antipsychotics in Tianjin, China

**DOI:** 10.1186/s12913-015-0819-y

**Published:** 2015-04-09

**Authors:** Xiaoning He, Jing Wu, Yawen Jiang, Li Liu, Wenyu Ye, Haibo Xue, William Montgomery

**Affiliations:** School of Pharmaceutical Science and Technology, Tianjin University, No 92 Weijin Road, Nankai District, Tianjin, China; Department of Clinical Pharmacy and Pharmaceutical Economics and Policy, School of Pharmacy, University of Southern California, Los Angeles, CA USA; Lilly Suzhou Pharmaceutical Company, Ltd., Shanghai, China; Eli Lilly and Company, Sydney, Australia

**Keywords:** Schizophrenia, Typical antipsychotics, Atypical antipsychotics, Resource utilization, Direct medical costs, China

## Abstract

**Background:**

It is uncertain whether the extra acquisition costs of atypical antipsychotics over typical antipsychotics are offset by their other reduced resource use especially in hospital services in China. This study compared the psychiatric-related health care resource utilization and direct medical costs for patients with schizophrenia initiating atypical or typical antipsychotics in Tianjin, China.

**Methods:**

Data were obtained from the Tianjin Urban Employee Basic Medical Insurance database (2008–2010). Adult patients with schizophrenia with ≥1 prescription for antipsychotics after ≥90-day washout and 12-month continuous enrollment after first prescription was included. Psychiatric-related resource utilization and direct medical costs of the atypical and typical cohorts were estimated during the 12-month follow-up period. Logistic regressions, ordinary least square (OLS), and generalized linear models (GLM) were employed to estimate differences of resource utilization and costs between the two cohorts. One-to-one propensity score matching was conducted as a sensitivity analysis.

**Results:**

1131 patients initiating either atypical (N = 648) or typical antipsychotics (N = 483) were identified. Compared with the typical cohort, the atypical cohort had a lower likelihood of hospitalization (45.8% vs. 56.7%, P < 0.001; adjusted OR: 0.58, P < 0.001) over the follow-up period. Medication costs for the atypical cohort were higher than the typical cohort ($438 vs. $187, P < 0.001); however, their non-medication medical costs were significantly lower ($1223 vs. $1704, P < 0.001). The total direct medical costs were similar between the atypical and typical cohorts before ($1661 vs. $1892, P = 0.100) and after matching ($1711 vs. 1868, P = 0.341), consistent with the results from OLS and GLM models for matched cohorts.

**Conclusions:**

The atypical cohort had similar total direct medical costs compared to the typical cohort. Higher medication costs associated with atypical antipsychotics were offset by a reduction in non-medication medical costs, driven by fewer hospitalizations.

## Background

Schizophrenia is a complex neurobehavioral disorder that adversely affects a broad range of psychological functions including thinking, perception, ideation, concentration, motivation, and judgment [1,2]. It affects approximately 1% of the population worldwide [3], and the prevalence in China was estimated at 0.78% during 2001 to 2005 [4]. Despite its relatively low prevalence compared to some other chronic diseases, schizophrenia imposes a significant burden on patients and their families due to its early age of onset, debilitating symptoms, and frequent relapses [5,6]. The economic burden of schizophrenia can be broadly divided into direct costs and indirect costs. Direct health care costs associated with the care of patients with schizophrenia, including the costs of medications, hospitalizations, diagnostic tests and medical procedures, and long-term care services have been reported to range from 1.5% to 3% of the total national health care expenditure in developed countries [5-8]. In addition, the burden of indirect costs, related to lost productivity due to unemployment, absence from work, and premature mortality have been estimated to be even greater than direct costs in many countries [8,9]. Consistent with studies from western countries, a regional study in China suggested that the economic burden for Chinese patients with schizophrenia was also considerable ($2586 per patient-year), of which 66% was due to indirect costs [10].

A cure for schizophrenia has not yet been found, but most patients’ symptoms can be improved with pharmacotherapy. Antipsychotic medications are the main stay of treatment used to manage acute psychotic exacerbations and as maintenance therapy to prevent relapse among patients with schizophrenia [11]. The first-generation antipsychotic medications, also called typical antipsychotics, were introduced in the 1950s. They not only provided therapeutic benefit for patients with schizophrenia but also carried the risk of important adverse events including extrapyramidal symptoms [12]. The introduction of the second-generation (so called atypical) antipsychotics, in the 1990s, was viewed as an improvement over the existing pharmacological options. Evidence from the literature has shown that atypical antipsychotics (including clozapine, olanzapine, and risperidone) are more efficacious for both positive and negative symptoms, and are associated with fewer extrapyramidal side-effects than typical antipsychotics; however, they are associated with greater increases in body weight and other metabolic parameters [13,14]. The atypical antipsychotics are generally recommended as first-line therapies in the clinical guidelines of many countries including China [1,2,14].

However, the utilization of branded atypical antipsychotics may have been restricted in the years following their launch due to the higher daily acquisition costs, compared to the typical antipsychotics, which are available as generic formulations [15]. Some studies have shown that the additional medication costs of the newer atypical drugs may be offset by reductions in other types of health care spending, which is known as the offset hypothesis [16]. It assumes that, in terms of antipsychotics, patients using atypical medications may have improved medication adherence and reduced rates of relapse, which are associated with intense use of health care resources, relative to typical medications. This can result in reductions in the use of other medical services such as hospitalizations and acute care facilities [17,18]. There is still a debate about whether the lower subsequent non-medication medical costs associated with the use of atypical antipsychotics are sufficiently large as to offset their greater upfront medication costs [19-21].

The present study was therefore carried out to evaluate the health service utilization and costs of treatment initiation with atypical antipsychotics in patients with schizophrenia in comparison with typical antipsychotics, as prescribed in the context of routine clinical practice using real-world claims data in China. The first objective was to explore whether initiation with atypical medications was associated with better treatment outcomes compared with typical medications, as measured by better medication adherence and reduced rates of hospitalization. Secondly, the study aimed to explore whether atypical initiators had lower non-medication related medical costs, and if they did, whether the reductions were enough to offset their higher medication costs — in other words, whether atypical antipsychotics represented a cost-effective alternative to typical antipsychotics, based on the principle that a better outcome was achieved for a similar or lower total cost.

## Methods

### Data source

This retrospective study used the Urban Employee Basic Medical Insurance (UEBMI) Claims of Tianjin from 2008 through 2010, which was obtained from the Tianjin Municipal Human Resources and Social Security Bureau through a formal request for research purposes. The UEBMI system, which is one of three basic medical insurance systems in China, was established in 1998 and covers employees and retirees in both the public and private sectors. The enrollees of the UEBMI in Tianjin included almost 5.1 million unique members by 2010, representing 51.7% of the registered residences in Tianjin city [22]. The analytical sample in this study was a random sample of 30% of all enrollees in the Tianjin UEBMI. With the establishment of the electronic outpatient records in 2008, the UEBMI database used in the present study included all inpatient, outpatient and prescription claims of the enrollees. This database provided patient-level demographic information combined with all the data relating to itemized medical service items and costs. Specifically, inpatient and outpatient service claims included type of service, date of service, units (days) of service, amount billed, and amount paid. Prescription drug claims identified the specific product dispensed, quantity, strength, the date the prescription was filled, and the associated drug acquisition cost. As the analysis for this study was carried out on an anonymized database, ethical approval was not required.

### Study sample

Adult patients (≥18 years of age) who had a schizophrenia-relevant diagnosis (ICD-10 code F20 supplemented by the Chinese description) and an initial prescription for an antipsychotic between April 1, 2008 and December 31, 2009 were included. The antipsychotic medications investigated in the present study comprised all the typical (chlorpromazine, perphenazine, fluphenazine, thioridazine, sulpiride, pipotiazine, haloperidol, droperidol, penfluridol) and atypical antipsychotics (clozapine, olanzapine, quetiapine, risperidone) reimbursed by UEBMI. The index date was defined as the date of patients’ first prescriptions following a 90-day washout period during which patients did not receive any antipsychotics. Atypical and typical cohorts were defined according to patients’ initial prescription regardless of their subsequent switching patterns. Patients who received both atypical and typical drugs at the index date or who were not continuously insured for 3 months before (baseline period) or for 12 months after the index date (follow-up period) were excluded.

### Measures

Psychiatric-related health care resource utilization and direct medical costs for the 12-month follow-up period were estimated. Medical claims under the primary diagnosis of ICD-10 of F00-F99 (supplemented by Chinese description) were identified as psychiatric related. Pharmacy resource utilization included the use of any antipsychotics and concomitant medications including anxiolytics, anticholinergics, antidepressants, sedative-hypnotics medications, and mood stabilizers. Adherence was assessed by measuring the days on antipsychotics, which were the total number of non-overlapped days covered by antipsychotics during follow-up period. Persistence with antipsychotics was defined as the time to discontinuation for any cause and calculated as the number of consecutive days from the index date to the first medication gap of >30 days [23]. Medical claims of psychiatric-related hospitalizations and outpatient visits were estimated as medical resource utilization. Among medical resource users, the number of hospitalizations, length of stay per hospital admission, and the number of outpatient visits were analyzed.

Psychiatric-related costs were calculated from the payer’s perspective including the payment paid by the insurance and the patients. Direct medical costs were calculated as the sum of medication costs and non-medication medical costs. Medication costs were composed of antipsychotic medications, concomitant medications, and other medications. Non-medication medical costs corresponded to the use of any other health care services except medications, including the costs of both inpatient and outpatient services. The costs of inpatient services were further broken down into non-medication treatment costs, laboratory and diagnostic costs, and the costs of other medical services. Specifically, non-medication treatment costs corresponded to the costs of any other treatment except medications, which included nursing, monitoring, psychotherapy, behavioral training and intervention, etc. Laboratory and diagnostic costs referred to the costs of physical examinations and biochemical tests. Costs of other medical services included blood transfusions, medical consumables, air conditioning and heating fees.

### Statistical analysis

Descriptive analyses were undertaken to evaluate differences in baseline characteristics and outcomes between the atypical and typical cohorts. Group comparisons were performed using two-sample t-tests for continuous variables, and chi-square tests or Fisher’s exact tests for categorical variables.

Multiple regression procedures were further employed to test for differences of resource utilization and costs between the two cohorts. Logistic regression models predicting the presence of any medical or concomitant pharmacy utilization in the follow-up year were developed. Ordinary least square (OLS) models and generalized linear models (GLM) with a log link and a negative binomial distribution were both used to estimate the differences in total costs and cost components. Negative binomial distribution was employed to take into account the over-dispersion of the cost data. Modified Park tests indicated that this specification was adequate relative to other types of distributions such as the Gamma or Poisson distribution [24]. The incremental effects between two cohorts in GLM models were obtained using the method of recycled predictions [25].

All regression models adjusted for patient characteristics and indicators of disease complexity at baseline. Specifically, the following types of patient-level variables were used as control variables in each model: demographics (age, gender, and working/retired), mental comorbidities (depression, anxiety, sleep disorder, and other mental diseases), concomitant medications (anxiolytics, anticholinergics, antidepressants, sedative-hypnotics, and mood stabilizers), mean number of psychiatric-related hospitalizations and outpatient visits and the total psychiatric-related direct medical costs during the prior 3 months.

Statistical analyses were conducted using STATA 12.0. A P-value of <0.05 was considered as statistically significant. All costs were converted to USD with an exchange rate in 2009 (6.83 CNY equal to 1 USD).

### Sensitivity analysis

In observational studies, selection bias can be a potential limitation in evaluating the outcomes of alternative interventions because patients are not randomly assigned to different therapies. Accordingly, a sensitivity analysis using propensity-score matching, which can control observable selection bias and render the two treatment cohorts more comparable, was conducted to test the robustness of the estimated outcomes. In the current study, the propensity score is a subject’s probability of receiving atypical antipsychotics on the observed covariates. It was calculated with a binary logistic regression which controlled for all independent variables in multivariate analyses. A greedy, one-to-one matching algorithm without replacement was employed to form pairs of atypical and typical cohorts. With the non-replacement technique, when an atypical patient is matched to a typical patient, both cases are removed from the pool. The caliper width was narrowed down progressively from 0.2*δ (0.2 of the standard deviation of the estimated propensity score) until all the baseline variables appeared with no significant differences [26]. Outcomes of matched pairs were compared using paired t tests for continuous variables, and McNemar’s tests for categorical variables. Standardized differences, which are independent of sample size, were also estimated to compare balance in measured variables between patients in the matched sample with those in the unmatched sample, and the imbalance defined as absolute value >0.1 [27]. Logistic regressions for resource utilization, OLS and GLM models for cost differences were also conducted for matched sample.

## Results

### Baseline characteristics

A total of 1131 patients who initiated therapy with atypical (N = 648) or typical (N = 483) antipsychotics with a prior 3-month washout period were identified (Table 1). The study sample had a mean age of early 50s (atypical vs. typical: 51.3 vs. 52.6, P = 0.081). More than half of the patients in both cohorts were women (55.1% vs. 55.3%, P = 0.950), while being retired was less common among the atypical cohort (52.2% vs. 58.4%, P = 0.037). During the baseline period, the percentages of patients diagnosed with mental comorbidities and patients treated with concomitant medications among the two cohorts were similar, except that the atypical cohort was less likely to be treated with sedative hypnotics (1.4% vs. 3.5%, P = 0.018). Anxiolytics appeared to be the much more commonly used (more than 11% for both cohorts) than other kinds of concomitant medications (<5%). With similar numbers of psychiatric-related outpatient visits at baseline (0.17 vs. 0.22, P = 0.332), the atypical cohort was associated with a lower frequency of hospitalizations (0.02 vs. 0.05, P = 0.028), which was further associated with statistically significantly lower direct medical costs ($20 vs. $42, P = 0.027). In the sensitivity analysis, the propensity-score matching process selected 400 patients initiated with typical antipsychotics who were the closest in propensity-score values to their counterparts in the atypical cohort. After matching, the two cohorts were comparable at baseline, and none of the differences between the two cohorts remained statistically significant both with regard to p-value and to standardized difference.

**Table 1:** **Baseline characteristics of atypical and typical cohorts before matching and after matching (N = 1131)**

**Variable**	**Before matching**				**After matching**			
	**Atypical cohort N = 648**	**Typical cohort N = 483**	**p-value**	**Standardized difference**	**Atypical cohort N = 400**	**Typical cohort N = 400**	**p-value**	**Standardized difference**
**Demographic characteristics**								
Mean age (in years) [Mean(SD)]	51.3 (13.0)	52.6 (11.9)	0.081	**−0.106**	52.1 (11.8)	51.7 (11.7)	0.144	0.035
Female [n(%)]	357 (55.1%)	267 (55.3%)	0.950	−0.004	226 (56.5%)	224 (56.0%)	0.715	0.010
Retired [n(%)]	338 (52.2%)	282 (58.4%)	**0.037**	**−0.125**	222 (55.5%)	228 (57.0%)	0.109	−0.030
**Concomitant medications [n(%)]**								
Anxiolytics	74 (11.4%)	57 (11.8%)	0.843	−0.012	18 (4.5%)	22 (5.5%)	0.541	−0.046
Anticholinergics	28 (4.3%)	15 (3.1%)	0.290	0.064	1 (0.3%)	1 (0.3%)	1.000	<0.001
Antidepressants	24 (3.7%)	22 (4.6%)	0.473	−0.043	1 (0.3%)	4 (1.0%)	0.375	−0.095
Sedative-hypnotics	9 (1.4%)	17 (3.5%)	**0.018**	**−0.138**	1 (0.3%)	3 (0.8%)	0.625	−0.071
Mood stabilizers	7 (1.1%)	6 (1.2%)	0.787	−0.015	2 (0.5%)	1 (0.3%)	1.000	0.041
**Mental comorbidities [n(%)]**								
Sleep disorder	38 (5.9%)	29 (6.0%)	0.921	−0.006	14 (3.5%)	10 (2.5%)	0.481	0.059
Depression	15 (2.3%)	16 (3.3%)	0.309	−0.060	1 (0.3%)	4 (1.0%)	0.375	−0.095
Anxiety disorder	8 (1.2%)	9 (1.9%)	0.462	−0.051	3 (0.8%)	2 (0.5%)	1.000	0.032
Other psychotic disorder^#^	6 (0.9%)	3 (0.6%)	0.740	0.035	1 (0.3%)	0 (0.0%)	1.000	0.071
**Resource utilization and cost (prior 3 months)**								
Mean number of hospitalizations	0.02 (0.15)	0.05 (0.25)	**0.028**	**−0.128**	0.01 (0.07)	0.01 (0.07)	1.000	<0.001
Mean number of outpatient visits	0.17 (0.58)	0.22 (0.83)	0.332	−0.069	0.08 (0.34)	0.05 (0.36)	0.298	0.071
Direct medical cost ($) [Mean(SD)]	20 (124)	42 (200)	**0.027**	**−0.129**	4 (45)	3 (46)	0.287	0.021

Continuous variables were compared using two-sample t-tests for unmatched samples and paired t-tests for matched samples; categorical variables were compared using chi-square tests or Fisher's exact tests for unmatched samples and McNemar’s tests for matched samples. P-values and standardized differences in bold indicates statistical significance.

^#^ Other psychotic disorder corresponded to any other mental disorders (ICD-10 F00-99) except sleep disorder, depression and anxiety disorder, which included mania, bipolar disorder, schizoaffective disorders, disorders of adult personality and behavior, mental retardation, etc.

### Descriptive analyses of health care resource utilization

The descriptive statistics describing the health care resource utilization and mental comorbidities achieved by the atypical and typical cohorts over the 12-month follow-up period are shown in Table 2. No statistically significant differences in antipsychotic utilization were found between the two cohorts, both in regard to adherence and persistence. Antipsychotic medications were taken for 139.1 days in the atypical group and 143.7 days in the typical group over the 12-month follow-up period, and the mean time to medication discontinuation for any cause among the two groups was 111.0 days and 110.2 days, respectively. Patients who received atypical antipsychotics at the index date were significantly less likely to use concomitant anxiolytic (73.5% vs. 85.3%, P < 0.001) and anticholinergic medications (33.3% vs. 65.4%, P < 0.001), and were more likely to take antidepressants (22.7% vs. 16.2%, P = 0.006) and sedative-hypnotics medications (39.5% vs. 25.3%, P < 0.001). In terms of medical resource utilization, the atypical cohort had a lower rate of psychiatric-related hospitalization compared with the typical cohort (45.8% vs. 56.7%, P < 0.001), with a similar number of hospitalizations (1.65 vs. 1.72, P = 0.291) and length of stay per admission (85.6 vs. 94.8, P = 0.094) among inpatient users. In contrast, patients started with atypical medications had a statistically significantly higher rate of psychiatric outpatient visits (80.1% vs. 65.8%, P < 0.001) and a higher number of visits among outpatient users (7.95 vs. 6.96, P = 0.044). Besides that, the atypical cohort appeared to have a higher rate of mental comorbidities across depression (17.0% vs. 10.8%, P = 0.003), anxiety disorder (11.9% vs. 7.7%, P = 0.020) and other psychotic disorders (5.4% vs. 2.7%, P = 0.025). The study results did not change much when the sensitivity analysis was conducted for the matched cohorts. Almost all estimators of health care resource utilization exhibited the same trend as in the core analysis. Compared to the unmatched sample, there was no statistically significant difference between atypical and typical cohorts for percentages of patients diagnosed with depression (14.3% vs. 9.8%, P = 0.052) or anxiety disorder (10.5% vs. 6.8%, P = 0.063) among the matched sample.

**Table 2:** **Resource utilization and comorbidities for atypical and typical cohorts during 12-month follow-up period**

**Variable**	**Before matching**			**After matching**		
	**Atypical cohort N = 648**	**Typical cohortN = 483**	**p-value**	**Atypical cohort N = 400**	**Typical cohort N = 400**	**p-value**
**Pharmacy resource utilization**						
**Use of antipsychotics [mean(SD)]**						
Days on of all antipsychotics	139.1 (109.0)	143.7 (109.2)	0.483	143.2 (110.7)	143.9 (108.0)	0.938
Time to all-cause discontinuation	111.0 (117.8)	110.2 (114.0)	0.910	113.2 (117.0)	109.0 (111.8)	0.606
**Use of concomitant medication [n(%)]**						
Any use of anxiolytics	476 (73.5%)	412 (85.3%)	**<0.001**	275 (68.8%)	342 (85.5%)	**<0.001**
Any use of anticholinergics	216 (33.3%)	316 (65.4%)	**<0.001**	143 (35.8%)	265 (66.3%)	**<0.001**
Any use of antidepressants	147 (22.7%)	78 (16.2%)	**0.006**	80 (20.0%)	57 (14.3%)	**0.028**
Any use of sedative-hypnotics	256 (39.5%)	122 (25.3%)	**<0.001**	163 (40.8%)	100 (25.0%)	**<0.001**
Any use of mood stabilizers	78 (12.0%)	52 (10.8%)	0.507	47 (11.8%)	42 (10.5%)	0.569
**Medical resource utilization** ^**#**^						
Any psychiatric hospitalization [n(%)]	297 (45.8%)	274 (56.7%)	**<0.001**	192 (48.0%)	231 (57.8%)	**0.002**
The number of psychiatric hospitalizations [mean(SD)]	1.65 (0.76)	1.72 (0.81)	0.291	1.65 (0.76)	1.71 (0.83)	0.487
Length of stay per hospitalization [mean(SD)]	85.6 (84.7)	94.8 (84.2)	0.094	88.3 (86.9)	92.9 (82.9)	0.473
Any psychiatric outpatient visit [n(%)]	519 (80.1%)	318 (65.8%)	**<0.001**	310 (77.5%)	261 (65.3%)	**<0.001**
The number of psychiatric outpatient visits [mean(SD)]	7.95 (7.26)	6.96 (6.25)	**0.044**	7.71 (6.89)	6.52 (5.70)	**0.027**
**Mental comorbidities**						
**Mean number of mental comorbidities [mean(SD)]**	0.72 (0.92)	0.54 (0.84)	**<0.001**	0.64 (0.87)	0.49 (0.80)	**0.012**
**Specific mental comorbidities** ^**##**^ **[n(%)]**						
Sleep disorder	131 (20.2%)	84 (17.4%)	0.231	70 (17.5%)	63 (15.8%)	0.514
Unspecified mental disorder	112 (17.3%)	75 (15.5%)	0.432	63 (15.8%)	59 (14.8%)	0.700
Depression	110 (17.0%)	52 (10.8%)	**0.003**	57 (14.3%)	39 (9.8%)	0.052
Anxiety disorder	77 (11.9%)	37 (7.7%)	**0.020**	42 (10.5%)	27 (6.8%)	0.063
Other psychotic disorder	35 (5.4%)	13 (2.7%)	**0.025**	23 (5.8%)	7 (1.8%)	**0.004**

Continuous variables were compared using two-sample t-tests for unmatched samples and paired t-tests for matched samples; categorical variables were compared using chi-square tests for unmatched samples and McNemar’s tests for matched samples. P-values in bold indicates statistical significance.

^#^ The number of psychiatric hospitalizations/length of stay per hospitalization was only for patients who had psychiatric hospitalizations during follow-up period, and the number of psychiatric outpatient visits was only for patients who had psychiatric outpatient visits during follow-up period.

^##^ Unspecified mental disorder (ICD-10: F99) corresponded to mental disorder which was not otherwise specified across ICD-10: F00-98. Other psychotic disorder corresponded to any other mental disorders (ICD-10: F00-99) except sleep disorder, unspecified mental disorder, depression and anxiety disorder, which included dementia, mania, bipolar disorder, schizoaffective disorders, disorders of adult personality and behavior, mental retardation, etc.

### Descriptive analyses of direct medical costs

Table 3 presents the descriptive results of psychiatric-related direct medical costs for study patients over the 12-month follow-up period. The atypical cohort had significantly higher medication costs ($438 vs. $187, P < 0.001) compared with the typical cohort, and the difference was mainly attributable to antipsychotics ($288 vs. $63, P < 0.001). As the result of much more outpatient visits, the costs of outpatient services for the atypical cohort were twice as much as the costs for the typical cohort ($10 vs. $5, P < 0.001). However, compared with the typical cohort, the mean annual non-medication medical costs for the atypical cohort were significantly lower ($1223 vs. $1704, P < 0.001), primarily driven by the lower psychiatric related costs of inpatient services ($1213 vs. $1699, P < 0.001). Despite the significant differences in almost all cost components, the unadjusted mean direct medical costs for the two cohorts were statistically similar (atypical: $1661, typical: 1892; P = 0.100). As for the matched sample in the sensitivity analysis, almost all costs components showed similar results to the core analysis, with the exception that the costs difference for concomitant medications became statistically insignificant ($61 vs. $40, P = 0.064). Consistent with the results of the unmatched sample, the atypical matched cohort incurred lower total costs in comparison with the typical matched cohort with a statistically insignificant difference ($1711 vs. $1868, P = 0.341).

**Table 3:** **Direct medical costs for atypical and typical cohorts during 12-month follow-up period (in 2009 US$)**

**Variable**	**Before matching**				**After matching**			
	**Atypical cohort N = 648**	**Typical cohort N = 483**	**Diff**	**p-value**	**Atypical cohort N = 400**	**Typical cohort N = 400**	**Diff**	**p-value**
**Total costs**	1661 (2224)	1892 (2465)	−231	0.100	1711 (2240)	1868 (2450)	−157	0.341
**Medication costs**	438 (626)	187 (337)	251	**<0.001**	409 (526)	172 (334)	237	**<0.001**
Antipsychotic medication	288 (431)	63 (216)	225	**<0.001**	268 (343)	64 (230)	204	**<0.001**
Concomitant medication	73 (266)	42 (98)	31	**0.014**	61 (194)	40 (99)	20	0.064
Other medication^#^	77 (197)	82 (213)	−5	0.680	81 (207)	68 (189)	13	0.344
**Medical costs**	1223 (2058)	1704 (2344)	−482	**<0.001**	1302 (2097)	1696 (2325)	−394	**0.011**
**Costs of inpatient services** ^**##**^	1213 (2061)	1699 (2346)	−486	**<0.001**	1292 (2099)	1692 (2327)	−399	**0.010**
Non-medication treatment costs	667 (1186)	992 (1443)	−325	**<0.001**	716 (1223)	977 (1422)	−262	**0.005**
Laboratory and diagnostic cost	254 (456)	388 (548)	−134	**<0.001**	278 (477)	393 (546)	−115	**0.001**
Other inpatient services	292 (539)	319 (476)	−27	0.384	299 (516)	321 (482)	−22	0.528
**Costs of outpatient services**	10 (28)	5 (26)	5	**0.004**	9 (25)	4 (11)	5	**<0.001**

Two-sample t-tests for unmatched samples and paired t-tests for matched samples. P-values in bold indicates statistical significance.

^#^ Other medication corresponded to any other medication except antipsychotic and concomitant medication, which included antihypertension medication, hypoglycemic medication, antibiotics, cardiovascular and cerebrovascular drugs, digestive system drugs, respiratory system drugs, antineoplastic drugs, etc.

^##^ Non-medication treatment costs corresponded to the costs of any other treatment except medication, which included nursing, monitoring, psychotherapy, behavior training and intervention, etc. Laboratory and diagnostic costs referred to the costs of physical examinations and biochemical tests. Costs of other medical services included blood transfusion, medical consumable, air conditioning and heating fees.

### Multivariate analyses

Logistic regression results for medical and concomitant pharmacy utilization are summarized in Table 4. Controlling for baseline characteristics, we found that the odds of being hospitalized during the follow-up period among the atypical cohort were still significantly lower than for the typical cohort (OR = 0.58, [95% CI, 0.44-0.75], P < 0.001). With respect to outpatient services, the atypical cohort was about twice as likely to have an outpatient visit as the typical cohort (OR = 2.19, [95% CI, 1.64-2.93], P < 0.001). Consistent with the unadjusted results (Table 2), patients initiating atypical medications were less likely to utilize concomitant anxiolytic (OR = 0.43, [95% CI, 0.31-0.60], P < 0.001) or anticholinergic medications (OR = 0.24, [95% CI, 0.18-0.31], P < 0.001), and were more likely to take antidepressants (OR = 1.64, [95% CI, 1.18-2.28], P = 0.003) and sedative-hypnotic medications (OR = 1.89, [95% CI, 1.45-2.46], P < 0.001). All regression models that were based on matched cohorts exhibited the same trend as the unmatched sample.

**Table 4:** **Regression-adjusted odds ratios on resource utilization between atypical and typical cohort (atypical vs. typical)**

**Variable**	**Before matching N = 1131**			**After matching N = 800**		
	**OR**	**95% CI**	**p-value**	**OR**	**95% CI**	**p-value**
**Medical service**						
Any psychiatric hospitalization	**0.58**	0.44-0.75	**<0.001**	**0.66**	0.49-0.89	**0.006**
Any psychiatric clinical visit	**2.19**	1.64-2.93	**<0.001**	**1.81**	1.32-2.50	**<0.001**
**Use of concomitant medication**						
Any use of anxiolytics	**0.43**	0.31-0.60	**<0.001**	**0.37**	0.26-0.53	**<0.001**
Any use of anticholinergics	**0.24**	0.18-0.31	**<0.001**	**0.26**	0.19-0.35	**<0.001**
Any use of antidepressants	**1.64**	1.18-2.28	**0.003**	**1.49**	1.01-2.20	**0.044**
Any use of sedative-hypnotics	**1.89**	1.45-2.46	**<0.001**	**2.11**	1.55-2.88	**<0.001**
Any use of mood stabilizers	1.11	0.75-1.64	0.605	1.09	0.69-1.72	0.706

Logistic regressions adjusted for baseline characteristics including demographics (age, gender and retired), mental comorbidities, concomitant medication, prior resource utilization and costs during baseline. Bold type indicates statistical significance.

Table 5 presents the marginal differences in OLS and GLM models of total costs and costs by type of service. Medication costs were significantly higher among the atypical cohort than the typical cohort (marginal differences: $247 in OLS; $242 in GLM per patient), which was mainly due to differences in the cost of antipsychotic medications (OLS: $217; GLM: $235). In terms of medical costs, patients started with atypical antipsychotics incurred statistically significant reduced costs of inpatient services (OLS: −$476; GLM: −$569) and increased costs of outpatient services (OLS: $5; GLM: $6). In the sensitivity analysis, consistent with the unmatched sample, the atypical matched cohort was associated with statistically significant decreased medical costs (OLS: −$399; GLM: −$450), which were more than enough to offset the increased medication costs (OLS: $238; GLM: $234). The total annual costs for the atypical cohort were significantly lower than the typical cohort in the GLM models (−$238, P = 0.031), while the differences were not statistically significant in the OLS models (−$229, P = 0.091) and regressions for the matched cohort (OLS: −$161; GLM: −$150).

**Table 5:** **Regression-adjusted cost differences during 12-month follow-up period between atypical and typical cohort (atypical vs. typical)**

**Variable**	**Before matching N = 1131**				**After matching N = 800**			
	**OLS**		**GLM**		**OLS**		**GLM**	
	**Diff**	**p-value**	**Diff**	**p-value**	**Diff**	**p-value**	**Diff**	**p-value**
**Total costs**	−229	0.091	**−238**	**0.031**	−161	0.329	−150	0.245
**Medication costs**	**247**	**<0.001**	**242**	**<0.001**	**238**	**<0.001**	**234**	**<0.001**
Antipsychotic medication	**217**	**<0.001**	**235**	**<0.001**	**204**	**<0.001**	**229**	**<0.001**
Concomitant medication	**30**	**0.016**	**22**	**<0.001**	21	0.052	**16**	**<0.001**
Other medication^#^	1	0.959	7	0.190	12	0.392	**15**	**0.005**
**Medical costs**	**−476**	**<0.001**	**−569**	**<0.001**	**−399**	**0.011**	**−450**	**<0.001**
**Costs of inpatient services** ^**##**^	**−481**	**<0.001**	**−592**	**<0.001**	**−404**	**0.010**	**−555**	**<0.001**
Non-medication treatment cost	**−315**	**<0.001**	**−401**	**<0.001**	**−269**	**0.004**	**−351**	**<0.001**
Laboratory and diagnostic cost	**−136**	**<0.001**	**−158**	**<0.001**	**−113**	**0.002**	**−131**	**<0.001**
Other inpatient services	−30	0.324	−39	0.056	−22	0.532	−29	0.229
**Costs of outpatient services**	**5**	**0.002**	**6**	**<0.001**	**5**	**<0.001**	**5**	**<0.001**

Regressions adjusted for baseline characteristics including demographics (age, gender and retired), mental comorbidities, concomitant medication, prior resource utilization and costs during baseline. Bold type indicates statistical significance.

^#^ Other medication corresponded to any other medication except antipsychotic and concomitant medication, which included antihypertension medication, hypoglycemic medication, antibiotics, cardiovascular and cerebrovascular drugs, digestive system drugs, respiratory system drugs, antineoplastic drugs, etc.

^##^ Non-medication treatment costs corresponded to the costs of any other treatment except medication, which included nursing, monitoring, psychotherapy, behavior training and intervention, etc. Laboratory and diagnostic costs referred to the costs of physical examinations and biochemical tests. Costs of other medical services included blood transfusion, medical consumable, air conditioning and heating fees.

## Discussion

To our knowledge, this study is the first comprehensive, retrospective comparison of the psychiatric-related health care resource utilization and direct medical costs for patients initiated with atypical or typical antipsychotics in China. The results suggested that patients initiated with atypical antipsychotics were significantly less likely to be hospitalized during follow-up period compared to patients initiated with typical antipsychotics, and less likely to use concomitant anxiolytic and anticholinergic medications, even though they demonstrated higher rates of mental comorbidities. As a result, the atypical cohort was found to incur lower medical costs relative to the typical cohort, which were sufficient to offset their higher medication costs.

Psychiatric-related hospitalization is often the consequence of a relapse or exacerbation of psychosis for patients with schizophrenia [28]. Consistent with some previous studies, a lower rate of psychiatric-related hospitalizations was observed among patients initiated with atypical medications in the present study, despite both cohorts displaying similar levels of medication adherence [29,30]. Our study may provide some evidence for the superiority of atypical antipsychotics in terms of reducing psychiatric hospitalizations, suggesting that they may have better efficacy and/or fewer or better tolerated side effects. Unlike inpatient services, the number of outpatient visits for the atypical cohort was significantly higher than the typical cohort. These results are similar to previous findings reported in the literature showing that atypical antipsychotic therapy resulted in higher resource utilization in outpatient care, which may indicate closer interactions between patients and psychiatrists and further lead to better adherence and lower relapse rates [31,32].

Overall the rates of antipsychotic medication persistence observed in this study were low, with a mean time to medication discontinuation for any cause among two groups of 111.0 days and 110.2 days, respectively, and approximately half the rate as that seen in similar naturalistic studies from the US [33,34]. This finding warrants further examination of the antipsychotic treatment patterns of patients with schizophrenia in China. Time to all-cause treatment discontinuation is considered a composite proxy measure of treatment efficacy, safety, and tolerability, and strategies to improve medication persistence have been shown to have the potential to improve patient outcomes [35].

Concomitant medications were widely used in our sample. Nearly 90% of patients received some kinds of psychiatric-related concomitant medications, which were similar with the findings from Nielsen et al. [36]. Anxiolytic medications were the most commonly prescribed concomitant medication among the two cohorts. They were prescribed less often in the atypical cohort despite a higher prevalence of anxiety disorders, which may confirm that atypical antipsychotics may be more effective than typical ones in controlling anxiety disorders [37,38]. The atypical cohort also, as expected, received significantly fewer anticholinergic medications, which may further support the lower risk of extrapyramidal side effects associated with this class of medications in clinical settings [13,39]. However, patients initiating atypical antipsychotics were significantly more likely to take antidepressants and sedative-hypnotics medications than the typical cohort. That partially could be the result of a high prevalence of mental comorbidities such as depression and sleep disorders in the atypical cohort.

Our results are consistent with the findings from previous studies that found that the use of atypical antipsychotics was associated with increased medication costs and decreased medical costs [29,40]. Both the descriptive and multivariate analyses showed that the higher medication acquisition costs among patients initiated with atypical antipsychotics were sufficiently offset by their lower medical costs, which is consistent with the findings of a study from Taiwan [29]. The results were confirmed by the sensitivity analysis after propensity-score matching, which suggests that the study results are relatively robust.

The results of this study need to be interpreted with care. Firstly, this study used claims data from the UEBMI insurance database, which includes both people who are employed and those who are retired. Individuals in this system may not reflect persons utilizing services in other systems of care. As such, these results may not be generalizable to the whole population with schizophrenia in Tianjin, China. Secondly, insurance claims data do not provide detailed patient-level information on clinical symptoms and disease severity. Although the results after propensity-score matching are similar to the unmatched results, unobserved confounders (e.g., severity of illness) may potentially affect the results. Thirdly, all atypical antipsychotics were considered as a homogenous group of drugs in our study, as were all typical antipsychotics. In reality, there are differences in the efficacy, effectiveness, and adverse-effect profiles between individual drugs within the same group [41,42]. Lastly, the 12-month follow-up period may not be long enough to observe changes in costs and outcomes with a chronic illness like schizophrenia. Studies with a longer-term follow-up period may be warranted.

## Conclusions

The present study estimated and compared the psychiatric-related health care resource utilization and direct medical costs associated with the initial prescription with atypical antipsychotics compared with typical antipsychotics in the treatment of schizophrenia in patients from Tianjin, China. Medication costs were higher for patients initiated with atypical antipsychotics during the 12-month follow-up. However, the atypical cohort was less likely to be hospitalized compared to the typical cohort, which in turn led to decreased medical costs. Initiation with atypical antipsychotics for patients with schizophrenia did not appear to increase the use of health care services as a whole, and it was associated with a reduction in medical costs that was sufficient to offset the higher medication costs for the atypical antipsychotics.
